# Developing a Personalized Meal Recommendation System for Chinese Older Adults: Observational Cohort Study

**DOI:** 10.2196/52170

**Published:** 2024-05-30

**Authors:** Zidu Xu, Yaowen Gu, Xiaowei Xu, Maxim Topaz, Zhen Guo, Hongyu Kang, Lianglong Sun, Jiao Li

**Affiliations:** 1 Institute of Medical Information Chinese Academy of Medical Sciences and Peking Union Medical College Beijing China; 2 School of Nursing Columbia University New York, NY United States; 3 Department of Chemistry New York University New York, NY United States

**Keywords:** knowledge graph, personalized food recommendation, geriatric nutrition, community, ubiquitous computing

## Abstract

**Background:**

China’s older population is facing serious health challenges, including malnutrition and multiple chronic conditions. There is a critical need for tailored food recommendation systems. Knowledge graph–based food recommendations offer considerable promise in delivering personalized nutritional support. However, the integration of disease-based nutritional principles and preference-related requirements needs to be optimized in current recommendation processes.

**Objective:**

This study aims to develop a knowledge graph–based personalized meal recommendation system for community-dwelling older adults and to conduct preliminary effectiveness testing.

**Methods:**

We developed ElCombo, a personalized meal recommendation system driven by user profiles and food knowledge graphs. User profiles were established from a survey of 96 community-dwelling older adults. Food knowledge graphs were supported by data from websites of Chinese cuisine recipes and eating history, consisting of 5 entity classes: dishes, ingredients, category of ingredients, nutrients, and diseases, along with their attributes and interrelations. A personalized meal recommendation algorithm was then developed to synthesize this information to generate packaged meals as outputs, considering disease-related nutritional constraints and personal dietary preferences. Furthermore, a validation study using a real-world data set collected from 96 community-dwelling older adults was conducted to assess ElCombo’s effectiveness in modifying their dietary habits over a 1-month intervention, using simulated data for impact analysis.

**Results:**

Our recommendation system, ElCombo, was evaluated by comparing the dietary diversity and diet quality of its recommended meals with those of the autonomous choices of 96 eligible community-dwelling older adults. Participants were grouped based on whether they had a recorded eating history, with 34 (35%) having and 62 (65%) lacking such data. Simulation experiments based on retrospective data over a 30-day evaluation revealed that ElCombo’s meal recommendations consistently had significantly higher diet quality and dietary diversity compared to the older adults’ own selections (*P*<.001). In addition, case studies of 2 older adults, 1 with and 1 without prior eating records, showcased ElCombo’s ability to fulfill complex nutritional requirements associated with multiple morbidities, personalized to each individual’s health profile and dietary requirements.

**Conclusions:**

ElCombo has shown enhanced potential for improving dietary quality and diversity among community-dwelling older adults in simulation tests. The evaluation metrics suggest that the food choices supported by the personalized meal recommendation system surpass autonomous selections. Future research will focus on validating and refining ElCombo’s performance in real-world settings, emphasizing the robust management of complex health data. The system’s scalability and adaptability pinpoint its potential for making a meaningful impact on the nutritional health of older adults.

## Introduction

### Background

With older population continues to grow globally [1], China leads with the largest older population proportion worldwide, expecting 28% of its population to be >60 years of age by 2040 [2]. An estimated 25.3% of China’s older adults are diagnosed with multiple chronic conditions (MCCs) [3]. Nutrition plays a critical role in healthy aging and contributes significantly to mitigating age-associated comorbidities [4]. However, according to the Chinese Longitudinal Healthy Longevity Survey results [5], 35% of the older adults (aged >65 years) is at risk of malnutrition [6]. This is largely attributed to inappropriate eating behaviors, particularly a lack of food diversity and poor diet quality [7], which can further lead to nutritional deficiencies and dietary disequilibrium [8,9], thereby negatively exacerbating MCCs and affecting overall well-being [6,10].

Currently, approximately 45% of the Chinese older adults (aged >65 years) are community dwellers (ie, outside nursing homes or hospitals) who live alone or with only one spouse in home environments [11,12]. They face challenges such as reduced physical function and altered sensory perceptions, which impact their dietary habits and nutritional intake [1,13,14]. These issues, along with cultural and literacy barriers [14-16], collectively reduce their adherence to a diverse, high-quality diet [17]. Several strides have been made in promoting healthy eating among community-dwelling older adults, primarily through health education and providing uniform nutrient supplements or food services [18,19]. However, these interventions often stick to the dietary guidelines for general populations. Their focus on adequate consumption of certain food groups and nutrients might not be applicable to older individual needs and preferences [16,20]. While some interventions offer flexibility by involving clinicians or nutritionists for personalized dietary recommendations or monitoring [21], the restricted professional and information resources are insufficient and not sustainable in addressing the community-dwelling older adults’ complex nutritional needs associated with MCCs [20,22].

Older adults’ eating behaviors are influenced by multiple factors, including physical and physiological factors, age-related issues, and social determinants [1,23]. Therefore, ideal diets for the older adults should be personalized, considering individual health, activity levels, and preferences, and guided by expert knowledge [16,23]. Simultaneously, addressing these needs effectively is a central objective of personalized food recommendation systems.

Table 1 categorizes the main types of food recommendation systems by focus area, applications, and approaches, highlighting their respective strengths and limitations.

**Table 1:** A summary of food recommendation system types, applied algorithm, and application scenarios.

Food recommendation system type	Application scenarios	Recommendation approaches	Reflections
Type 1: user preferences focused [24-26]	Web-based food ordering platforms and recipe websites	Content based, knowledge based, collaborative filtering, and hybrid approach	Tailored to user tastes for high satisfaction; it may neglect nutrition and variety beyond past preferences.
Type 2: nutritional needs focused [27-29]	Specialized meal plans for patients with specific health conditions and diet therapy	Constraint based, goal-oriented recipe, and knowledge based	It addresses health and dietary needs but may not match user tastes and depends on precise health data.
Type 3: balancing preferences and needs [30,31]	Home meal planning and health-conscious food apps	Constraint based, content based, and knowledge based	Balances enjoyment with health, yet managing this balance poses a complexity.

Type 1, focused on user preferences, recommends dishes similar to those previously enjoyed by the user, using collaborative filtering and content-based algorithms for item rating and user profile similarity computations. It is most applicable to personal dining and web-based food platforms, where individual tastes drive the selection process. Type 2 emphasizes nutritional needs, often using constraint-based approaches to recommend health-conscious meals, catering to users with specific dietary requirements or health conditions. Type 3 highlights a balance between user preferences and nutritional needs. This is often applied in scenarios such as home meal planning or health-focused food apps. Despite a diverse focus, personalized food recommendation systems generally share similar algorithms, including collaborative filtering, content-based, and knowledge-based systems. Collaborative filtering algorithms use user ratings to match and recommend items liked by similar users. They excel in user preference prediction but face the cold start problem with small sample sizes. Content-based systems, which focus on item features and user history, achieve personalization but may limit diversity by predominantly suggesting previously preferred items. Knowledge-based systems make recommendations based on explicit user requirements and deep domain knowledge. They are effective in scenarios with sparse user data and particularly offer high customization in well-defined use preference settings or specialized domains such as health care. In practice, a hybrid approach is more often applied to mitigate individual limitations. Specifically, in community-dwelling older adults food recommendations targeting a relatively small number of users, the integration of nutritional and health considerations is paramount. This context indicates that knowledge-based recommendations are suitable choices, with domain expertise ensuring dietary appropriateness. A complementary role can be played by content-based systems, which align recommendations with individual taste profiles while together delivering nutritionally sound and appealing food choices.

A food knowledge graph (FoodKG) can be viewed as a graph-structured database aiming to organize food-related entities (data nodes), attributes, and the connected edges (relations) between them in a network of interconnected entities [32,33]. They serve as integral components of knowledge-based food recommendation systems, organizing interconnected food-related entities to facilitate contextually appropriate dietary suggestions [33]. Personalized food recommendation systems based on a FoodKG aim to synthesize comprehensive personal information to customize dietary recommendations, which has the strength of providing sharable and uniform representations of food and nutrition knowledge to address the problem of information silos across multiple sources [33,34]. While these systems have been implemented successfully across various sectors of the food industry, such as personalized diet therapy planning for patients with specific diagnoses and food and restaurant recommendations to the general population [32,34-40], their application often lacks specificity for community-dwelling older adults, a demographic with unique dietary needs due to multimorbidity and personal preferences. Currently, there is a scarcity of FoodKG and corresponding KG-based food recommendation systems specifically designed to meet the unique dietary needs and health considerations of this demographic. The absence of targeted data, such as actual meal consumption data and primary data on these older adults’ health status and dietary preference, leads to this significant gap. This challenge highlights the necessity of developing a customized FoodKG-based focused on the community-dwelling older adults, incorporating primary data that capture the nuances of their health status and dietary choices. Consequently, a personalized recommendation system built on this FoodKG should effectively manage the complex interplay between dietary restrictions due to older adults’ MCCs and personal dietary preferences, which is a key aspect of community-based daily nutrition support targeting the older adults [31].

Finally, considering the alignment of current food recommendation systems with their target users, the prevalent method of offering a wide variety of dish options may not be suitable for the older adults. Community-dwelling older adults generally have lower nutrition literacy compared to younger users, who are the primary audience for most web-based food or restaurant recommendations. In addition, they do not have nutrition knowledge support similar to what hospitalized patients receive from clinical nutritionists. This lack of nutrition knowledge makes it challenging for them to select from abstract food recommendations, understand appropriate portion sizes, and create nutritionally balanced meals [34,39,41,42]. Addressing the unique dietary needs of community-dwelling older adults requires a nuanced approach that considers their lower nutrition literacy and lack of nutrition knowledge support compared to other demographics. Digital tools, such as web-based assistants, offer potential for user-friendly support, yet their integration into food recommendation systems for the older adults is still emerging. Thus, a deeper study of FoodKG-based meal recommendations is warranted to meet the personalized nutritional needs of the older adults in their daily diets. Accordingly, we identified three key questions: (1) how to address the complex nutritional requirements associated with multimorbidity, (2) how to integrate personal dietary preferences into rigorous nutritional principles, and (3) how to ensure user-friendliness for older adult users.

### Objectives

**Figure 1:**
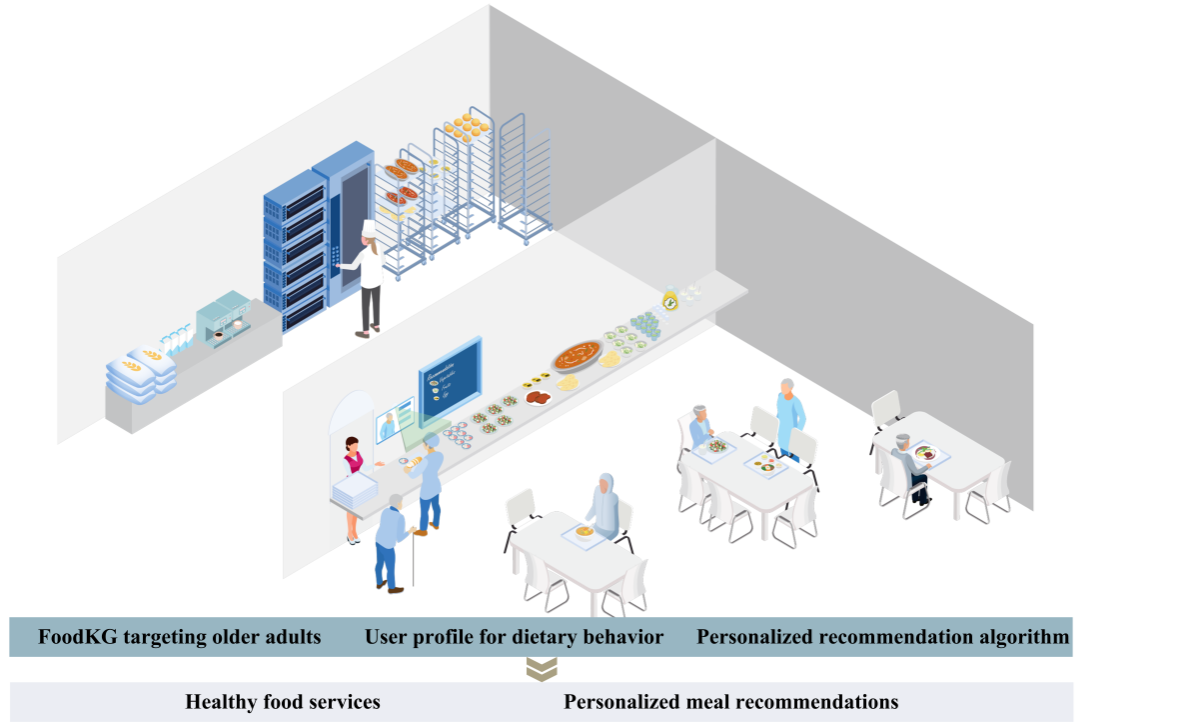
A scenario landscape of ElCombo application in personalized meal recommendation for community-dwelling older adults. FoodKG: food knowledge graph.

In response to these considerations, the primary objective of this study is to propose ElCombo, short for “Elderly Combo,” a personalized meal recommendation system specifically designed for community-dwelling older adults. Figure 1 illustrates the conceptualized application scenario of the ElCombo system among community-dwelling older adults, depicting the workflow from the FoodKG construction to the delivery of personalized meal recommendations. ElCombo aims to offer eating choices in the form of meal combos, which are combinations of several dishes. It considers both disease-related nutritional requirements and personal dietary preferences of community-dwelling older adults while also providing a user-friendly delivery mode. The secondary aim is to examine the effectiveness of ElCombo in improving the diet diversity and diet quality for community-dwelling older adults using the primary data collected in this study. Our overarching aim is to assist these older adults, especially those in home environments with limited access to professional clinical support, in making informed decisions faced with overwhelming information presented by advanced technologies, consequently contributing to promoting healthy aging.

## Methods

Guided by a user-centered design concept, ElCombo was designed based on the needs and knowledge acquired from real-world data, combined with the recommendation outputs in the format of packaged meals, that is, combinations of several dishes, to reduce the mental loads of community-dwelling older adult users.

### ElCombo System Architecture

**Figure 2:**
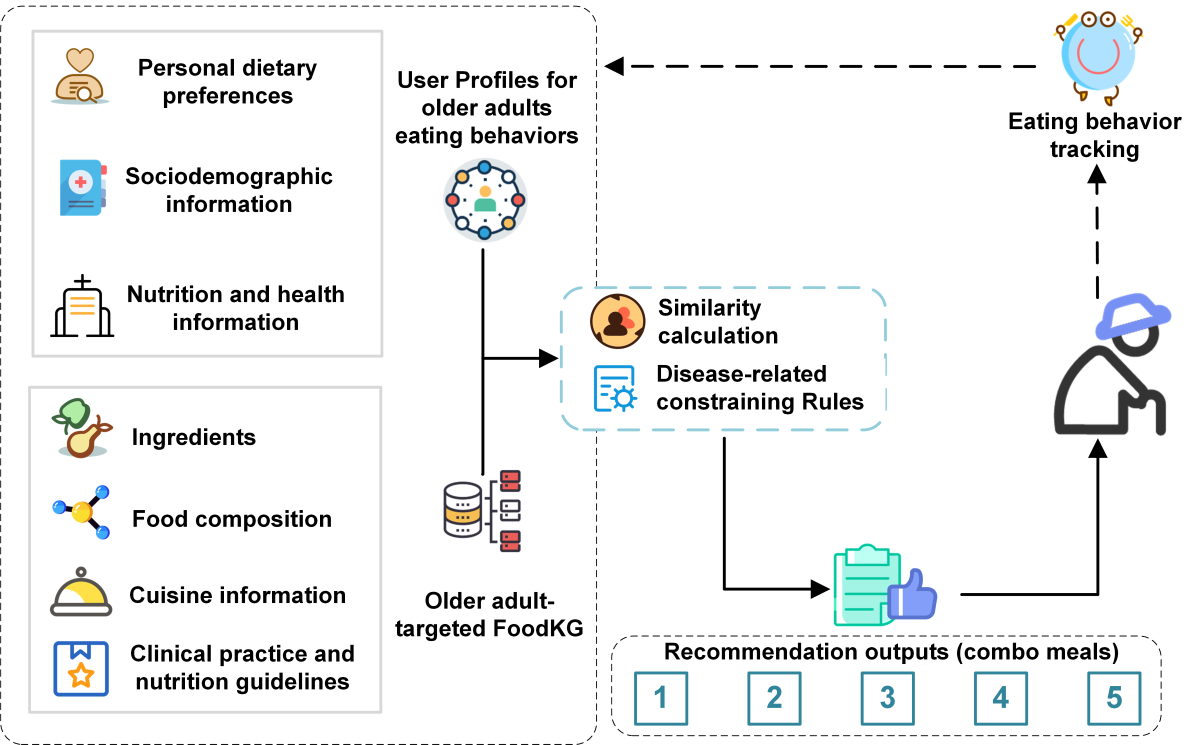
Overview of ElCombo’s architecture for older-adult-specific food recommendation, including knowledge graph and algorithm modules.

Figure 2 presents the architecture of personalized food recommendation system targeting community-dwelling older adults. As shown in the diagram, ElCombo is consisted of the following three key modules:

1. FoodKG targeting community-dwelling older adults, which served as a knowledge base to collect and store information on candidate dishes, relevant ingredients, nutrients, common geriatric diseases and disease-related constraints. Since the FoodKG involved the experts’ knowledge of food composition and food intake criteria informed by the diagnosis, it also supported regulating the whole recommendation pipeline.
2. The user profile data set targeting older adults’ dietary behaviors, which captured personal needs related to disease-specific nutritional requirements and dietary preferences. It includes demographics, nutrition and health status, and dietary preference information for community-dwelling older individuals. This user profile data set served as a key input for a nutritional recommendation approach. It leveraged data from survey results of community-dwelling older adults, along with eating history tracked by the Internet of Things (IoT) system deployed at the canteen (if applicable).
3. The personalized meal recommendation algorithm, which generated tailored meal plans for community-dwelling older adults, combined nutritional expert knowledge and individual dietary needs using similarity calculations and rule-based filters to create user-friendly packaged meals. The algorithm was driven by 3 factors: nutritional context for initial food filtering, short-term goals for daily meal generation based on user preferences and needs, and long-term goals for adjusting recommendations according to eating history.

### Initial Data Preparation

The initial steps necessary to prepare the data to be used in meal recommendation generation were aimed at identifying appropriate data sets to achieve two intermediate goals: (1) constructing the FoodKG and (2) building user profiles related to older adults’ dietary behaviors.

#### Data Sources

A total of 6 data sources were identified to contribute to the FoodKG construction in 3 aspects. First, food and recipe data were gathered from 2 sources: historical eating choices from local canteens for community-dwelling older adults and various recipe websites. Second, food composition and nutrient information were sourced from a specific database, the Chinese Food Composition Table and the Dietary Nutrient Reference Intake for Chinese Residents [43,44]. Finally, common geriatric diseases and corresponding nutrition restrictions were collected from textbooks and clinical guidelines related to the included diseases.

#### Data Collection and Preprocessing

To make use of the information from food and recipe websites, we first converted the data from its original semistructured HTML format on web pages into a more structured, tabular format that was suitable. This was done using the Scrapy framework by Zyte (formerly Scrapinghub) in a Python 3.8 environment, where we extracted specific information from web pages, including dish names, ingredients, seasonings, cooking methods, and flavors. Once extracted, we conducted the data preprocessing by converting these details into a structured table format. The transition from web page representations of dishes to their tabular format in data sheets is shown in Figure S1 in Multimedia Appendix 1 [45-51], using “Stir-fried Spinach” as an example.

To prepare for the real-word recommendation, we also included information on 42 dishes informed by the tracked eating history of community-dwelling older adults. These dishes were already formatted into tables with details, such as dish names, ingredients, and cooking methods. We manually removed 30 dishes that were receptive to the dishes obtained from websites, then finalized a list of 180 unique dishes for building our FoodKG, as explained in Figure S2 in Multimedia Appendix 1. In the second phase of data preprocessing, we standardized the weights and ratios for the dishes, ingredients, and seasonings, as presented in Table S1 in Multimedia Appendix 1. The nutrient content of each food item was calculated based on the standard amount of nutrients present in every 100 grams of the ingredients involved. This step linked all relevant data, including dishes, ingredients, ingredient categories, and nutrients, and prepared them for the next step in our recommendation process.

We took 3 main steps to gather and preprocess the data indicating the nutritional requirements of older adult individuals with geriatric diseases. The first step involved locating 30 common geriatric diseases across 12 human systems from a textbook of geriatric medicine [45]. Next, we identified statements within textbooks and clinical guidelines that describe the relationships between these diseases and various ingredients, ingredient categories, and nutrients. These guidelines cover a broad spectrum of common geriatric management topics, such as cardiovascular and metabolic disease management, diabetes management, nutrition therapy for patients with cancer, and dietary guidance for patients with stroke [46-52].

The third step was the extraction of entities and relations from these statements. Regular expressions were applied, considering the standardized and well-formatted statement expressions in the textbooks and clinical guidelines. We developed an exhaustive list of expressions of relations and corresponding variations to systematically capture the relations and their triples in the statements. The outputs of this process include the main categories of relations, the triples containing the relations, examples of the statements extracted, and the sources from which these statements were derived (Table S2 in Multimedia Appendix 1). This methodical approach helped organize the data on disease-nutrient interactions, facilitating the development of our nutritional recommendation framework for the older adults.

#### FoodKG Construction

**Figure 3:**
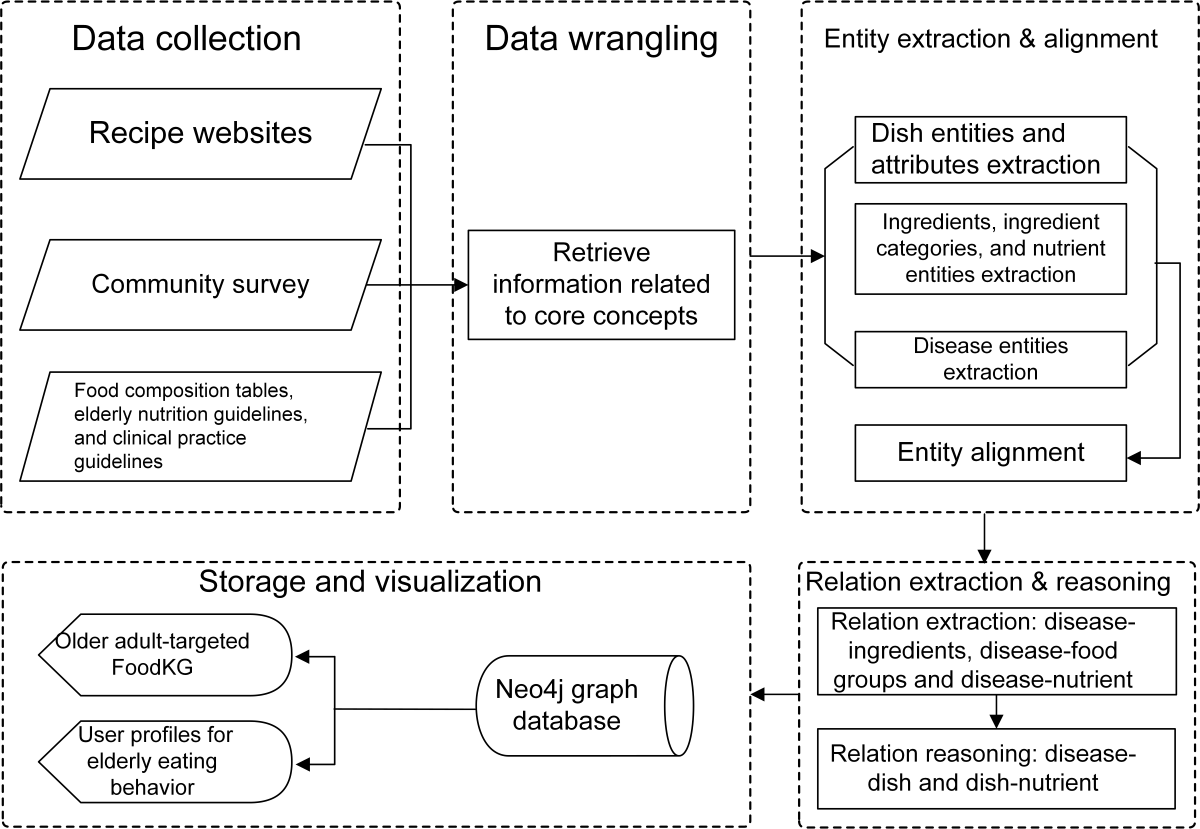
Food knowledge graph construction pipeline.

FoodKG construction generally followed a top-down pipeline to align with authoritative guidelines and expert opinions (Figure 3). The FoodKG construction process can be divided into four steps: (1) schema design, (2) data preparation, (3) knowledge extraction and reasoning, and (4) knowledge storage and representation.

Drawing on prior literature and expert opinions, we initially defined 5 central concepts to shape the framework of the FoodKG: dishes, ingredients, categories of ingredients, nutrients, and diseases. We also established their respective attributes and interrelationships. As depicted in Multimedia Appendix 1 (Figure S3 and Tables S3 and S4 in Multimedia Appendix 1), the 5 pivotal concepts are interconnected. A “dish” refers to any edible cuisine composed of various “ingredients,” which include raw materials and seasonings, thus establishing a “consist_of” relation between “dishes” and “ingredients.” These “ingredients” are grouped into different “ingredient categories” in accordance with dietary guidelines, and relevant “nutrients” are identified from food composition tables. “Diseases” are incorporated as the final crucial concept, given their significant relevance to nutritional support for the older adults.

Following the aforementioned 5 pivotal concepts, we obtained food, nutrition, and disease-related data from various sources and preprocessed it into a standard tabular format. We used the Scrapy framework to crawl “dishes” entities from the “older adults” section of recipe websites. Simultaneously, we extracted website data tagged with “cooking method” and “flavor” labels, serving as the attributes of “dish” entities. We included a “dish category” as an additional attribute to facilitate meal package recommendations. Furthermore, we extracted “main components” and “seasonings” labels attached to “dish” entities as “ingredients” and identified their interrelationships. We sourced “disease” entities from clinical practice guidelines and textbooks on common geriatric diseases. This process yielded a corpus of words, phrases, and sentences describing relationships between diseases and ingredients, food groups, and nutrients. During data preprocessing, we standardized the weight of each dish category and the proportions of seasonings and food contained. Specifically, we set a standard sodium resolution in each dish at 0.8%-1.0% to reduce the risk of hypertension in community-dwelling older adults.

We resolved potential name variations and ambiguities through knowledge fusion, implementing entity alignment, and named entity disambiguation. We eliminated “dish” entity duplicates based on name similarity, included ingredients, and cooking methods. We set an ingredient similarity threshold of 85% and kept the most frequently occurring combinations as the standard for the corresponding “dish” entity. After removing desserts and snacks, we finalized 180 unique “dish” entities associated with unique sets of “ingredients” entities and attributes. We conducted the entity alignment of “ingredients” and “nutrient” entities according to the standard names listed on food composition tables. This resulted in 112 “ingredient” entities and 27 “nutrient” entities. We directly acquired “ingredient categories” and “disease” entity names from food nutrient intake instructions and textbooks. For these, we executed the entity extraction process by importing the standard names of 20 “ingredient categories” entities and 30 “disease” entities, bypassing the entity alignment.

Guided by the top-down design, the relationships between the entities of “dishes,” “ingredients,” “ingredient categories,” and “nutrients” were established based on natural interactions. We identified 3 types of relationships among “diseases” and “nutrients,” “ingredients,” and “ingredient categories,” namely discourage, restrict, and recommend. This categorization was synthesized from textbooks, clinical practice guidelines, and expert opinions. We used rule-based relation extraction to identify disease-related triples, relying on a relation dictionary developed from our predefined relationship types, followed by manual checks to ensure accuracy.

**Figure 4:**
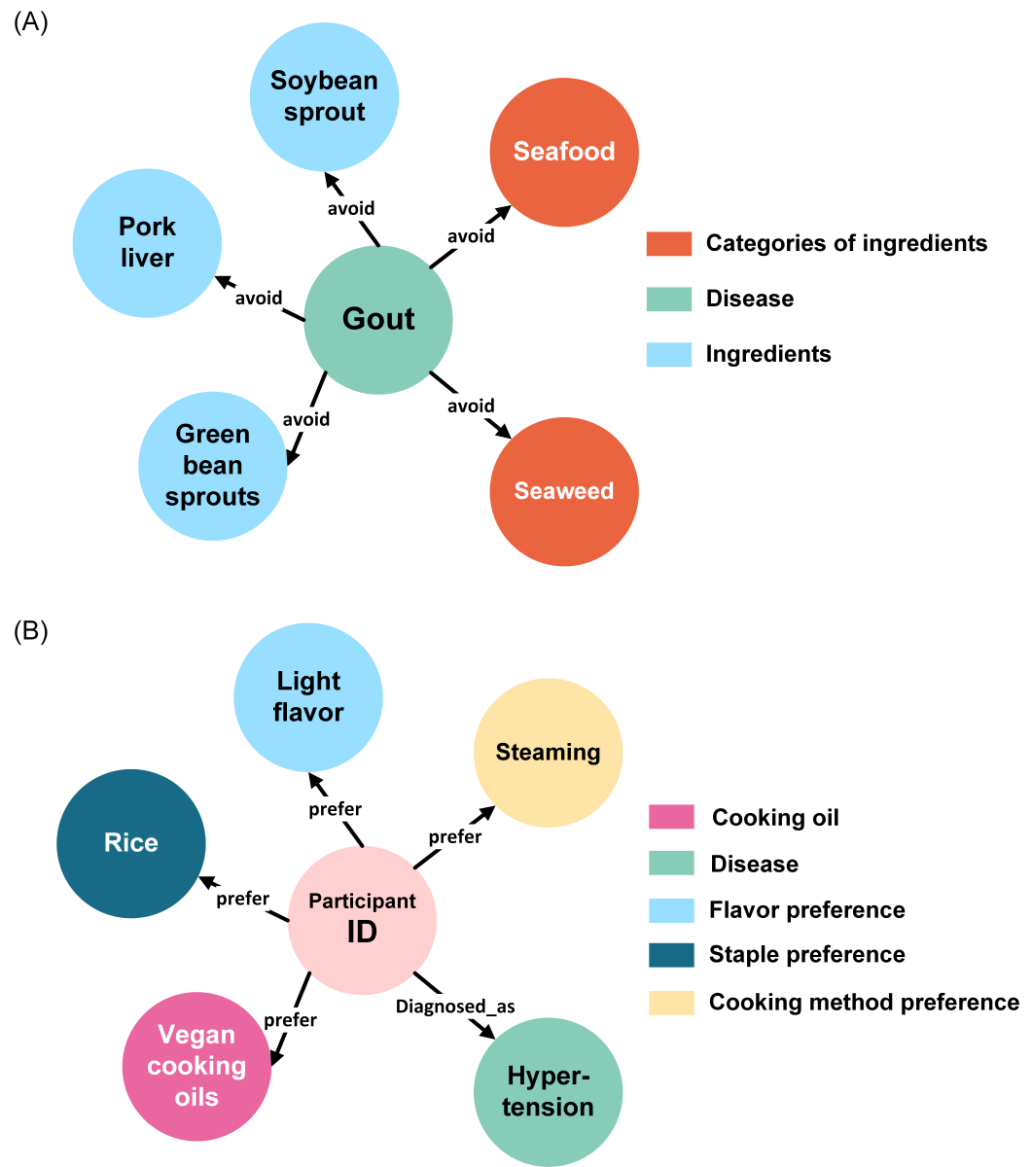
Visualizing disease-diet connections and individual health profiles in FoodKG. (A) Mapping “gout” disease to relevant dietary ingredients and categories; (B) Older adult individual profile with hypertension; dietary preferences and interconnections depicted.

Despite our extraction efforts, the relationships between “dishes” and “nutrients,” “dishes” and “ingredient categories,” and “diseases” and “dishes” remained undefined. We hypothesized that these relationships could be inferred from the existing entities and relations within our constructed FoodKG, denoted as KG=<E,R,T>, and the relation path P. Here, E and T represent the set of entities, R represents the set of relations, and the edges in R link 2 nodes to form a triple (h, r, t)∈T. We sought to generate a new triple that did not exist in the knowledge graph, denoted as G=([h, r, t] | h∈E,r∈R,t∈T, [h, r, t]∉G). This allowed us to infer new relationships and their properties that were missed during the initial extraction process. We performed this knowledge reasoning using a first-order logic reasoning method. For example, we used the formula Contain (IngredientA, NutrientA, M)∧Consist_of (DishA, IngredientA, P)⇒Contain(DishA, NutrientA, Q) to determine the relationship between a specific dish and nutrient entities (ie, nutrient weights contained in a dish unit) by accumulating nutrients contained in different ingredients making up the dish according to proportions. The formula: Discourage (DiseaseA, IngredientA) ∧ Consist_of (DishA, IngredientA)⇒ Discourage (DiseaseA, DishA) was used to determine relationships between specific diseases and dishes. Figure 4A shows the disease entity “gout” and its relations to relevant ingredients and ingredient categories.

The finalized older adult-targeted FoodKG comprised 369 entities, 6 relationships, and 10,071 triples, stored in a Neo4j graph database. This data structure is a directed labeled graph consisting of nodes, edges, and labels. As illustrated in Figure S4 in Multimedia Appendix 1, the entities of dishes (orange), ingredients (blue), and food category (red) function as nodes. An edge connects a pair of nodes and represents the interrelations, while the labels of the edge further specify the nature of these relations.

#### User Profile Establishment

Parallel to FoodKG, we built user profiles for older adults eating behaviors to capture the individual characteristics of older adult target users, thus facilitating recommendation customization. Similarly, following a top-down workflow, we designed the architecture of user profiles, including 3 dimensions: sociodemographic, disease and health conditions, and dietary preferences. We used the survey data to obtain real-world status and actual needs. A total of 96 community-dwelling older adults in Hangzhou, China, consented to participate in the survey. A total of 16 items were included in the questionnaire. Among them, the dietary preference–related items were extracted from Chinese Longitudinal Healthy Longevity Study (2018) questions, and the sociodemographic and health condition items were created based on the medical records in the primary care center.

The user profiles were also stored in a Neo4j graph database to support entity and relation queries as a prerequisite for food recommendation. As shown in Figure 4B, the pink node represents the entity of a community-dwelling older adult individual, the green nodes with the label “hypertension” represent their diagnosis of disease, and other nodes represent the dietary preference from different perspectives. The edges between different nodes represent the properties of interrelations.

#### Personalized Meal Recommendation Algorithms

**Figure 5:**
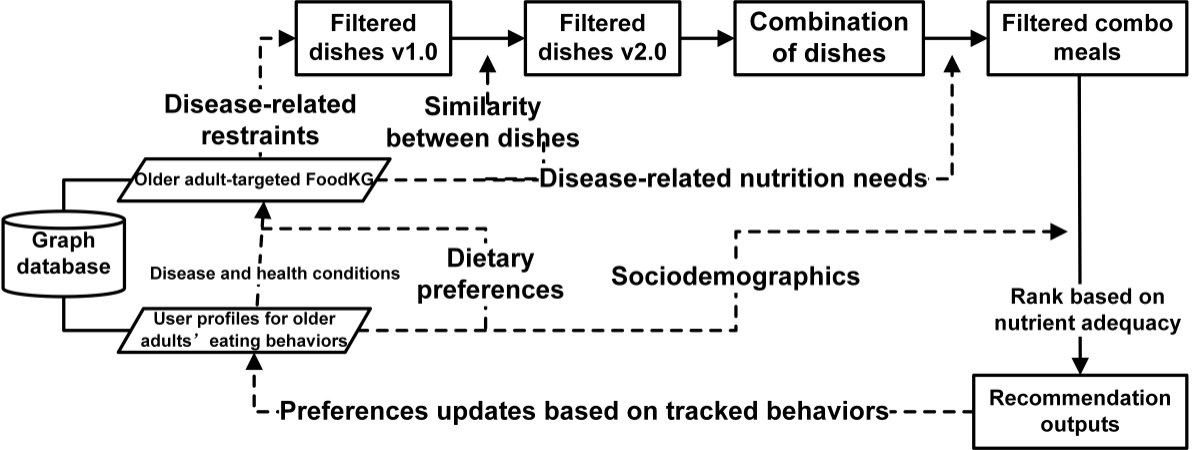
Dual-stage personalized meal algorithm: dish generation and combo meal recommendations.

As shown in Figure 5, the personalized meal recommendation algorithm has 2 main components: candidate dish generation and combo meal recommendation.

The generation of candidate dishes can be divided into 2 phases: initial filtering and dish reranking. The initial filtering step aimed to eliminate dishes with ingredients unsuitable for older adult individuals due to disease-related dietary restrictions. It began with the application of disease-related restraints, informed by the information in the FoodKG and the user profiles for older adult dietary behavior. After this initial filtering, the algorithm further refined the selection of dishes by considering personal dietary preferences. This step considered the following two scenarios:

1. Hot boot for community-dwelling older adults with tracked eating history: we used a method to rank dish candidates based on their similarity to previously selected dishes. This ranking is derived by assessing the ingredient composition of dishes. As ingredient content in dishes was quantified as continuous numerical data, cosine similarity was adopted to assess the similarity between dishes.
2. Cold boot for community-dwelling older adults without tracked eating history: we identify a reference older adult with a closely matching user profile. The assumption is that older adults with similar profiles will have similar dietary preferences. The similarity between profiles is calculated based on their features. Because the user profile features were recoded as categorical variables, the Jaccard similarity measure was used for comparison. In addition, as the recommendation duration increases, the preferences and recommendation preferences could be continuously updated based on tracked eating behaviors.

Combo meal recommendations focused on combining various dishes into meal combos that are nutritionally balanced and cater to the diverse dietary preferences and economic statuses of the older adult community. The process involved the following three key steps:

1. Initial combo meal creation: multiple dishes from different categories were involved to form initial combo meal candidates. This selection was guided by the goal of creating diverse combinations of dish categories at varying price levels, ensuring accessibility for community-dwelling older adults of different socioeconomic statuses. Table 2 lists all possible combinations of different types of dishes.
2. Nutritional constraints assessment: the algorithm then evaluated the combo meals against the individual’s nutritional needs. Meals that did not adhere to the requirements specified in nutrient guidelines in terms of ingredient or ingredient category restrictions would be filtered out.
3. Final combo meal ranking: the remaining meals were ranked based on their nutrient adequacy. This was measured by the number of nutrients that satisfied the criteria outlined in the nutrition guidelines for Chinese older adults [44,53].

**Table 2:** Dish combinations considered in the meal recommendation algorithm.

Price	Dish counts	Dish categories
High	4	1×animal-derived dish+1×vegan dish+1×staples+1×soup
High	4	2×omnivorous dishes+1×staples+1×soup
Intermediate high	3	1×animal-derived dish+1×vegan dish+1×staples
Intermediate high	3	2×omnivorous dishes+1×staples
Intermediate	2	1×animal-derived or omnivorous dish+1×complete dish
Low	2	1×vegan dish+1×complete dish

The detailed processes and calculations for candidate dish filtering and combo meal generation are provided as pseudocode in algorithms 1 and 2, located in Multimedia Appendix 2. The corresponding formulas for each calculation step are also included.

### Validation Study

#### Overview

In this study, we aimed to evaluate the impact of the ElCombo meal recommendation system on the dietary behaviors of community-dwelling older adults using a single-arm before-and-after design. To ensure safety and minimize risks, we will conduct a preliminary simulation test with the primary data from older participants. The effectiveness of the intervention will be assessed based on changes in diet quality and diversity, measured using the China Elderly Dietary Guidelines Index (CDGI-E) [52,54], and the dietary diversity score (DDS) [55,56].

#### Study Setting and Participants

To examine our hypothesis in validation study, we conducted a primary data collection among community-dwelling older adults in Hangzhou City, Zhejiang Province, China, from July 15 to July 21, 2021. All participating community-dwelling older adults and research team members were consented by the Institute of Medical Information, the Chinese Academy of Medical Sciences, and the Peking Union Medical College. See details in the Ethical Considerations section.

We view this validation study as a single-arm before-and-after study. The power calculation is based on the primary outcome measure—DDS. The sample size was calculated according to the repeated-measures formula [10,43,53]: 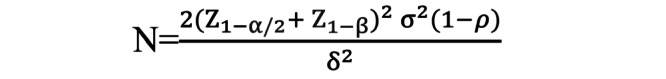 Considering a type I error rate of 5% (2-sided), a power level of 80%, the smallest detected meaningful difference (δ) of 2.0 based on expert opinion, a correlation coefficient (ρ) among measures of 0.5, and an equal SD (σ) of 6.0 were selected according to relevant studies [53,57]. It was estimated that the desired number of included participants at baseline should be set at a minimum of 71 for each arm. Consequently, a total of 89 community-dwelling older individuals were expected to recruit at baseline, considering an attrition rate of 20% [58-60].

Participants were selected using a convenience sampling method from community health service centers in Hangzhou. Older individuals were eligible if they were (1) aged >60 years, (2) able to communicate independently, (3) able to make independent food choices, (4) able to access community sites to receive interventions, and (5) able to take meals independently. The exclusion criteria included (1) being diagnosed with cognitive impairment, indicated by a Mini Mental State Examination score less than 24, or (2) having dietary restrictions strictly mandated by medical prescriptions due to the advanced severity of chronic conditions. Recruitment efforts involved posting flyers at community health service centers, particularly during regular physical examinations of older adult individuals.

#### Data Collection

The data collection for this validation study encompasses three major components:

- Demographic information, including age, sex, economic status, health care coverage, and education level, was collected through a basic information questionnaire designed by the research team.
- Health and nutritional status and general health status data, such as self-reported medical history, allergies, height, and weight, were gathered through physical measurements and basic information questionnaires. Levels of physical activity and risk of malnutrition and frailty were assessed through established questions, including International Physical Activity Questionnaire, Mini Nutritional Assessment-Short Form, and the short 5-question assessment of fatigue, resistance, aerobic capacity, illnesses, and loss of weight (FRAIL) scale.
- Dietary behavior: 2 approaches were provided to collect the dietary behaviors of the community-dwelling older adults. A dietary habits questionnaire extracted from the CLHILS questionnaire was mandatory for all participants to help understand their dietary habits and preferences. In addition, an IoT system has been deployed in the community canteen since 2020. This system automatically tracks the older residents’ dietary behaviors, offering a more objective and precise measure of their dietary choices.

The methods of data collection are detailed in Table 3, which provides a clear breakdown of the data dimensions, items, and the respective collection methods.

**Table 3:** Overview of demographic, dietary, and health and nutrition data collection among community-dwelling older adults.

Data dimensions	Data items	Collection method
Demographics	Sex, age, geography, living arrangements, and monthly household income	Basic information questionnaire designed by the team
Dietary behavior	Dietary diversity Diet quality	IoT^a^ system log (DDS^b^) IoT system log (CDGI-E^c^)
Health and nutritional status	BMI Level of daily physical activities Allergies and medical history Risk of malnutrition Risk of frailty	Basic body measurement (body weight scale and height rod) Questionnaire (IPAC^d^) Basic information questionnaire designed by team Questionnaire (MNA-SF^e^) Questionnaire (FRAIL^f^)
Personal dietary preferences	Cooking method preference, staple food preference, common oil preference, and taste preference Dish preference, if available	Questionnaire (CLHLS^g^) [61] or IoT system log

^a^IoT: Internet of Things.

^b^DDS: dietary diversity score.

^c^CDGI-E: China Elderly Dietary Guidelines Index.

^d^IPAC: International Physical Activity Questionnaire.

^e^MNA-SF: Mini Nutritional Assessment-Short Form.

^f^FRAIL: the short 5-question assessment of fatigue, resistance, aerobic capacity, illnesses, and loss of weight.

^g^CLHLS: Chinese Longitudinal Healthy Longevity Survey.

#### Simulated Interventions

In the simulated intervention phase of our study, we used the ElCombo meal recommendation system to provide personalized dietary advice and healthy food services to community-dwelling older adults (development details are provided in previous sections). This system was driven by 2 key databases: a dietary behavior user profile database constructed from primary data collected from the older participants and a FoodKG constructed for older adults, using historical dining behavior data from older residents and menus from the community canteen, with a total of 180 dish entities, 112 ingredient entities, 20 ingredient type entities, 27 nutrient entities, and 30 disease entities. The main scenario considered was the lunch hour, with the daily energy intake estimated to be divided among 3 meals at a 3:4:3 ratio.

Participants were provided with 30 days of simulated meal recommendations, offering 5 options for each weekday. These recommendations were tailored according to the availability of their historical eating behaviors. Specifically, we categorized participants into 2 groups based on their recorded eating history frequency (≥5 times) in the IoT system. The “tracked group,” with an eating history of no less than 5 instances, received initial recommendations based on the similarity between their frequently chosen dishes and candidate dishes. The nutrient considerations were also included. For those with <5 eating history records, the “untracked group,” recommendations were generated by comparing their profiles to those of similar individuals with >5 recorded eating behaviors. This approach ensured personalized and relevant suggestions for each group. We followed the recommending principles designed in the “Personalized Meal Recommendation Algorithms” section, considering that the participants randomly took one of the recommended meals generated by the ElCombo system.

#### Outcome Measuring

In the outcome measurement phase, we focused on evaluating the changes in diet diversity and diet quality of the community-dwelling older adults over a 1-month intervention period to determine the effectiveness of the ElCombo meal recommendation system. As a simulated intervention, all data regarding participants’ food selections were generated and stored locally. We retrieved this data from our repository to score the dietary scales for our analysis. For the comparison analysis, both groups set comparators using data from the tracked group. However, we compared the tracked group’s recommendations to their historical eating patterns over time. For the untracked group, comparator metrics were calculated based on the tracked group’s autonomous choices, in which we assumed the tracked and untracked groups had similar overall averages and distributions. In addition, case analyses were conducted to examine the system’s ability to tailor recommendations to the individual characteristics of community-dwelling older adults.

For dietary diversity, we used the DDS [62], a measure that reflects the variety of food categories in the participants’ diets, ranging from 0 to 9. The nonrepetitive counts of food categories contained in the combos are accumulated as the DDS. Previous studies have proved that DDS is a concise and straightforward dietary diversity indicator for older populations [55,62-64]. The higher the DDS, the higher the dietary diversity the lunch combos.

For diet quality assessment, the CDGI-E was used [54], with a range from 0 to 110. CDGI-E was developed based on Chinese dietary guidelines for seniors and Food Guide Pagoda, which was released in 2016 [52], serving as a diet quality index in large population-based diet surveys among older adults [54,55]. CDGI-E can be used to examine whether the experts experience and knowledge have been well incorporated into the recommendation system. The scoring functions of DDS and CDGI-E are provided in Multimedia Appendix 2.

### Ethical Considerations

This study received ethics approval from the Institute of Medical Information, the Peking Union Medical College, and the Chinese Academy of Medical Sciences (approval number IMICAMS/01/21/HREC). The participants provided written informed consent for both primary data collection and subsequent analyses. To protect the participants’ privacy, deidentification was conducted by removing direct personal identifiers; essential demographic details such as age and sex were securely retained for meal recommendation purposes. Personal records were stored separately, ensuring access only to authorized health care professionals and investigators. As a token of appreciation for their time and contribution, participants were compensated with daily necessities and health education materials valued at approximately US $15 for the initial assessment.

### Statistical Analysis

For descriptive analysis, continuous variables were presented as means with SDs or medians with IQRs, while categorical variables were expressed as percentages. The comparative analysis assessed diet quality and diversity changes over a 30-day intervention using a paired 2-tailed *t* test for the tracked group and an independent *t* test for the untracked group. In addition, a visualization report was generated, illustrating the outcome changes with 95% CIs for each time point. All analyses were performed using Python (version 3.8), with statistical significance set at a *P* value of <.05 (2-sided).

## Results

### Participants Characteristics

A total of 112 community-dwelling individuals were screened, among whom 96 eligible individuals were included for further data collection and analysis. Of these 96 participants, 34 (35%) had tracked eating history behaviors >5 times, and 62 (65%) had tracked eating behaviors <5 times. In total, 2 databases were incorporated into the validation procedure: an older adult–targeted FoodKG containing 180 dishes (refer to the details in the Methods section) and dietary-related user profiles contributed by 96 older participants.

### Overview of the Older Adult Participants and Involved Data Sets

Baseline characteristics of the older adult participants, including their demographics and general health status, are presented in Table 4.

**Table 4:** Characteristics of the community-dwelling older adult participants (N=96).

Characteristics			Participants, n (%)
**Sex**			
	Male	51 (53)	
	Female	45 (47)	
**BMI (kg/m^2^)**			
	≤19.9	2 (2)	
	20.0-26.9	66 (69)	
	≥27.0	27 (28)	
**Age (years)**			
	60-64	14 (14)	
	65-79	66 (69)	
	≥80	16 (17)	
**Diagnosis of chronic conditions**			
	≥3	36 (38)	
	2	24 (25)	
	1	31 (32)	
	0	5 (5)	

More comprehensive information regarding their socioeconomic status, medical history, and dietary preferences is available in Table S1 in Multimedia Appendix 3. In total, 96 participants were aged between 61 and 90 years, with an average age of 70.93 (SD 7.08) years and 45 (47%) being female. Particularly, we provided a deeper investigation into the MCCs of our older participants. Notably, >60% (59/96) of participants experienced ≥2 chronic conditions simultaneously and 38% (37/96) had ≥3 coexisting chronic conditions. The 3 most prevalent diseases were hypertension (58/96, 59%), arthritis (30/96, 31%), and diabetes mellitus (23/96, 24%).

For the older adult–targeted FoodKG, Table S5 in Multimedia Appendix 1 presents all contained entities of 180 dishes, 112 ingredients, 20 ingredient categories, 27 nutrients, and 30 diseases, which supported the personalized meal recommendations. The details regarding dish category, flavor, cooking methods, and contained ingredient distribution are provided in Figure S5 in Multimedia Appendix 1.

### Comparative Analysis

We divided the 96 older participants into a tracked group (n=34) and an untracked group (n=62), according to whether they had >5 eating history behaviors. Following this, a dual-step comparison analysis was conducted to examine the effectiveness of the simulated meal recommendation intervention. First, a paired 2-tailed *t* test and an independent *t* test analysis were, respectively, conducted to compare the DDS and CDGI-E scores before the intervention and after the intervention in both groups. As presented in Table 5, for both groups, CDGI-E and DDS scores were reported to be statistically significantly higher in the recommended meals compared to autonomous selections (*P*<.001).

**Table 5:** Diet quality and diversity score improvement with meal recommendations versus autonomous selections.

Group and indicators		Autonomous, mean (SD)	Recommended, mean (SD)	*t* test (*df*)	*P* value
**Tracked (eating history ≥5 times)**					
	CDGI-E^a^	25.66 (0.61)	29.51 (0.68)	24.54 (33)	<.001
	DDS^b^	3.73 (0.12)	4.9 (0.16)	28.3 (33)	<.001
**Untracked (eating history <5 times)**					
	CDGI-E	25.66 (0.61)	28.05 (51)	14.45 (37.96)	<.001
	DDS	3.73 (0.12)	4.58 (0.61)	27.72 (47.04)	<.001

^a^CDGI-E: China Elderly Dietary Guidelines Index.

^b^DDS: dietary diversity score.

**Figure 6:**
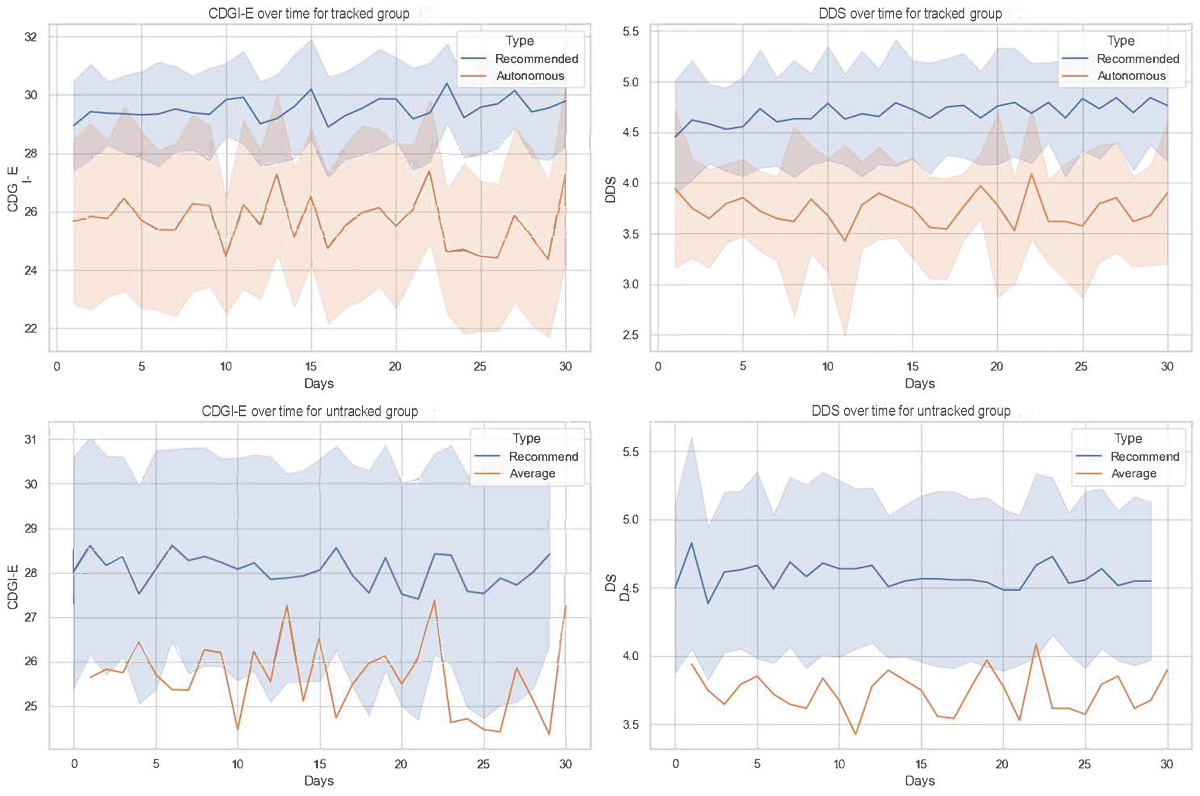
Visualization of 30-day simulation outcomes showing time evolution of China Elderly Dietary Guidelines Index (CDGI-E) and dietary diversity score changes. DDS: dietary diversity score.

To assess the changes in diet quality and diversity across 2 groups during the simulated intervention period, we also assessed the overall variations in CDGI-E and DDS over 4 weeks. Figure 6 illustrates time-dependent shifts in dietary quality and diversity within this period. The date range was plotted on the x-axis, while the y-axis showed the values of CDGI-E and DDS. The blue lines represent the average outcome indicators of the recommended meals, while the orange lines signify those of the autonomously selected meals. Particularly, the orange line was marked as “average” for the untracked group to represent the assumption mentioned above. Given that multiple recommendations were provided as alternatives, the 95% CIs of the outcome indicators are also depicted as shaded areas.

From the line charts, we identified 3 preliminary findings. First, the outcome indicators of recommendations (blue lines) are generally higher than those of autonomous selections, demonstrating that the meals recommended by ElCombo are superior in diet quality and diversity compared to the older adults’ independent choices. Second, the outcome indicators for the recommended meals display less fluctuation in both CDGI-E and DDS lines than the autonomous selections, indicating greater stability. This suggests that our proposed FoodKG recommendation system can help individuals maintain a consistently high-quality and diverse diet, which might otherwise be challenging to achieve independently. Finally, the blue lines indicate an increase in CDGI-E and DDS scores, underlining ElCombo’s potential to automatically optimize the recommended outputs over time.

### Representative Case Study

**Figure 7:**
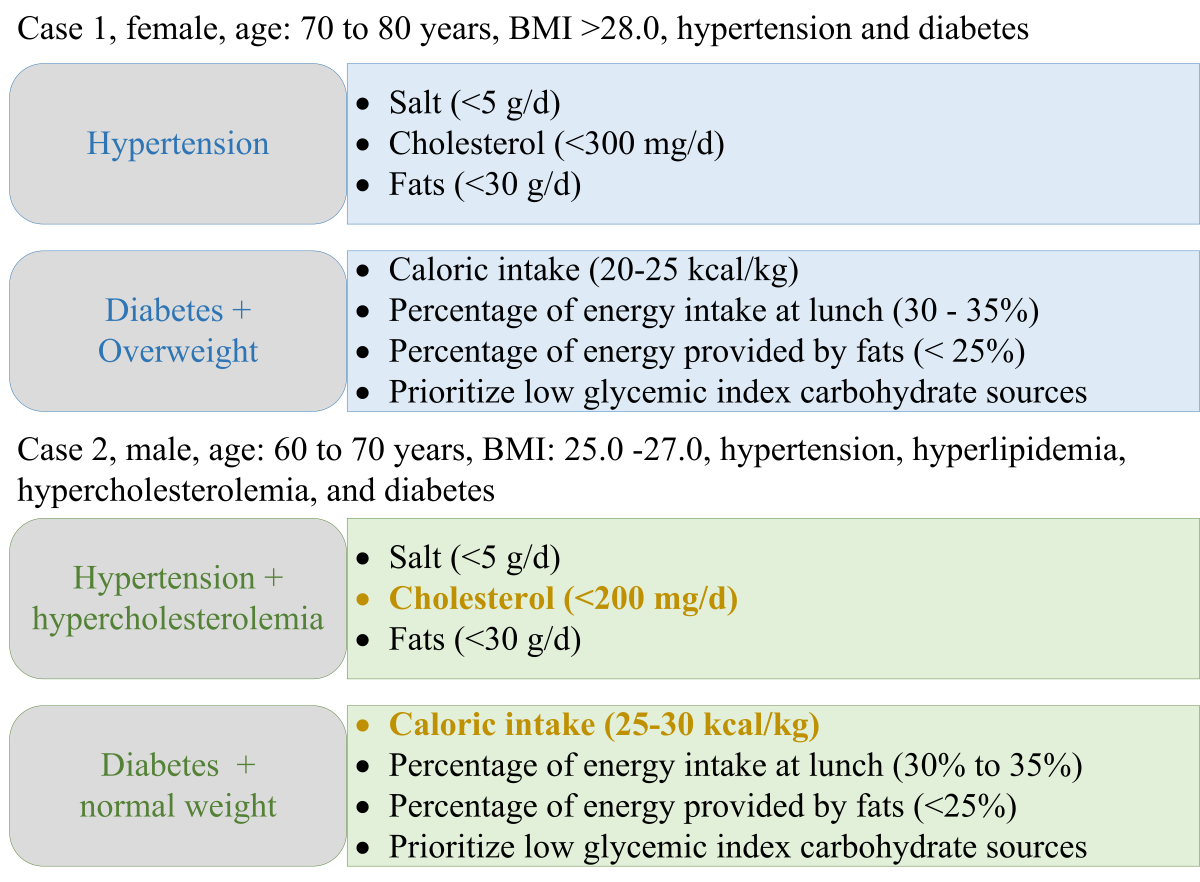
Health profiles and nutritional needs linked to the multiple chronic conditions of case study subjects.

We also conducted 2 case studies to dissect the ElCombo workflow aimed at older adult individuals. This allowed us to understand the potential benefits of a FoodKG-based meal recommendation system more deeply. We randomly selected 2 community-dwelling older adult individuals from the above described cohort for our study—one with a tracked eating history and the other without. Figure 7 displays the health profiles and nutritional needs associated with the MCCs of the 2 case study participants. We also provide the final week’s meal recommendations in Multimedia Appendix 4, which include specific dishes and their respective diet quality (CDGI-E) and diversity (DDS) scores. In addition, for the individual with a recorded eating history, we provide a comparison between recommended and autonomous meal choices to enhance the intuitive understanding of the personalized recommendations’ impacts.

Our first case study focused on a female participant in her 70s with a BMI >28.0 kg/m^2^, diagnosed with hypertension and diabetes. Her user profile data included a preference for salty flavors, a staple diet based on rice, and light daily physical activity. Adhering to the designed framework, we began by filtering out candidate meals that failed to meet older adults’ nutrition guidelines, particularly those related to her health conditions. Specifically, we imposed limits on salt, cholesterol, and oil content, dictated by “restrict” relations for these food entities. Her diabetes diagnosis and daily physical activity level also dictated energy intake (20 to 25 kcal/kg), energy proportion from lunch (30% to 35%), and energy proportion from fat (<25%). We generated the top 5 meal candidates per weekday based on nutrient adequacy ranking. These candidates adhered to both disease-related nutrition requirements and nutrition guidelines for older adults.

Upon comparing the recommended meals with those the subject selected independently over a week (5 weekdays), we noticed the increased diversity of dishes in the recommended meals. The recommended combos contained 17 dishes, compared to 9 dishes in the combos she selected. Our recommendations also provided more variety in staples and soups. This led to a marked increase in diet diversity and quality. Furthermore, the recommended meals had a high proportion of lower-glycemic-index carbohydrates such as whole grains, reflecting the tailored recommendation rules for her health conditions.

Our second case study examined a male participant in his 60s with a BMI between 25.0 kg/m^2^ and 27.0 kg/m^2^, diagnosed with hypertension, diabetes, and dyslipidemia. He preferred lighter flavors and rice-based meals. As his eating behavior history was unavailable, we used a reference older adult individual (case 1) with the highest Jaccard similarity of 0.7. Recommendations for the male subject were further refined based on the reference individual’s most frequently selected dishes. Health-related restrictions about diabetes, hypertension, and dyslipidemia helped us set limits on cholesterol (<200 mg/day) and energy intake (20 to 25 kcal/kg). We excluded those meals that did not meet disease-related nutrition requirements, resulting in 282 suitable meals out of the initial 708 candidate meals recommended by ElCombo.

Our system generated 17 unique dishes in various combinations for a 5-day meal plan, with a repetition rate of only 25%. Fewer meat-based dishes were recommended to the male participant than to the female participant, possibly due to the cholesterol-related preferences identified in his user profile. Despite variations in the recommended dishes, both meal sets demonstrated similar diet diversity and quality. This suggests that ElCombo can effectively generate diverse, high-quality meal recommendations for community-dwelling older adult individuals. This is achievable with minimal reliance on eating history while maintaining a keen focus on personal health status.

## Discussion

### Principal Findings

Healthy eating is crucial in mitigating MCCs and promoting healthy aging [1,65]. However, finding suitable solutions that cater to the specific needs of community-dwelling older adults remains a challenge [4]. In this study, we developed ElCombo, a personalized meal recommendation system, with specific attention to the unique challenges community-dwelling older adults face. We demonstrated the significant promise of ElCombo in improving the dietary behavior of community-dwelling older adults, particularly in terms of diet diversity and quality. This system offers a highly tailored and adaptable solution to the dietary challenges faced by this demographic. ElCombo represents a responsive approach to the evolving dietary habits of older adult users, ensuring that recommendations remain relevant and effective.

The development of our system involved 3 key steps: constructing a FoodKG for nutritional and disease-related knowledge support, establishing user profiles to match individual needs with food intakes, and developing a recommendation algorithm that allows updates based on dietary behavior changes. The use of automated technologies in our system makes it a cost-effective approach to individual dietary management [23]. Our experiments reveal that ElCombo outperforms autonomous dietary choices, with older adults showing improved performances in diet diversity and quality, as measured by DDS and CDGI-E. These findings underscore ElCombo’s effectiveness as a tool for promoting healthier dietary habits and enhancing health outcomes among community-dwelling older adults. Its time-awareness allows the recommendations to adapt to the dietary needs of community-dwelling older adults with MCCs.

### Strengths and Implications

Compared to other food recommendation systems, ElCombo has unique strengths and innovations stemming from its focus on the daily dietary needs of community-dwelling older adult individuals. Our research focus and designated approach are backed by distinctive data sources. Specifically, we collected data on the eating choices of community-dwelling older adults from menus in community canteens. In addition, we gathered primary data on their health status and dietary preferences to ensure a more rigorous and reliable validation study. This approach effectively balances the need for nutrient intake that meets disease-related requirements with the flexibility to cater to personal preferences, offering a more sustainable impact on older adults’ dietary health. Unlike previous systems that primarily offered guidance on specific food groups or nutrients [44], ElCombo provides more comprehensive and practical meal-based advice for daily eating scenarios [42,66]. This highlights a key feature of ElCombo—user-friendliness, which displays a variety of well-organized meals that integrate different dishes instead of a lengthy list of individual food items.

Although some existing food recommendation systems have previously offered meal-based suggestions [26,28,31], ElCombo stands out with its more comprehensive and tailored considerations for older adults. It prioritizes the complex health conditions common among the older adults, particularly those with MCCs. This has led to the development of a rigorous knowledge base and recommendation algorithm, enabling the system to address the broad spectrum of nutritional needs. In addition, ElCombo excels for its attention to food culture. Unlike systems that focus solely on main ingredients [31,67], ElCombo includes a detailed analysis of side ingredients and seasonings, ensuring a more comprehensive nutrient composition and precise recommendations. Finally, ElCombo demonstrates high compatibility with diverse socioeconomic statuses by offering diverse combinations with different dish types and quantities to suit varying food budgets.

The older-adult–targeted FoodKG built in this study, combining expert knowledge with user profiles and reasoning rules, enables ElCombo to handle individual case queries effectively. Similar to the findings from previous work, the knowledge-based food recommendation approach proves reliable in this study. The reliance on a rule-based recommendation pipeline in ElCombo is justified by its ability to manage complex interactions between food constraints and nutritional requirements, providing stable and streamlined meal recommendations.

Transforming complex clinical guidelines into detailed initiatives in community settings and home environments enhances the effectiveness of elderly care informed by abstract expert knowledge, leading to a profound impact on public health [53,68]. Accordingly, the potential benefits of our proposed system, which is tailored for daily meal recommendations for community-dwelling older adults, can be both durable and adaptable. Its methodology can be extended to other self-management practices among these community-dwelling older adults, including medication adherence, physical activity, and mental wellness programs. This personalized recommendation approach, characterized by comprehensive knowledge support and sensitive adaptation to individual needs, is especially beneficial for older adult individuals living with MCCs, helping them achieve a higher quality of life in their aging years.

### Limitations and Future Work

Despite its promise, the system does have limitations and areas for future development. The evaluation experiment was conducted in a simulation setting with a retrospective study design; also, the present system was designed to provide advice on recommended intake rather than actual intake [23,69]. These 2 concerns raise questions about the real-world effectiveness of our system. Future work will address this by conducting real-world clinical trials to evaluate the system’s effectiveness, acceptability, and user experience [70]. In addition, we recognize that the current data extraction efficiency from large corpora is a challenge, especially considering the complex health conditions and prescriptions often associated with older adults. In response, we plan to enhance our methodology by implementing deep neural networks for named entity recognition and relation extraction, thereby increasing the expansion speed of FoodKG [33]. This improvement will provide a more nuanced approach to addressing the unique health and prescription needs of the older adults.

### Conclusions

In this study, we developed and evaluated ElCombo, a personalized meal recommendation system designed for Chinese community-dwelling older adults. Our findings in simulated environments revealed that ElCombo holds the promise to effectively improve the dietary diversity and quality of the community-dwelling older adults, as reflected by the increase in DDS and CDGI-E scores. While these results are promising, they are preliminary and situated within a controlled, simulated context. The true effectiveness of ElCombo to enhance dietary practices and potentially impact health outcomes among the aging population remains to be established through rigorous real-world clinical trials. Consequently, future research is dedicated to conducting more comprehensive feasibility and effectiveness evaluations among community-dwelling older adults to confirm the system’s efficacy and refine its ability to process complex health data effectively. The adaptability and preliminary cost-effectiveness observed in this study suggest that ElCombo has the potential to be a valuable tool in improving the dietary health of older adult individuals, pending further validation.

## Appendix

Multimedia Appendix 1 Food knowledge graph data preparation.

Multimedia Appendix 2 The personalized meal recommendation algorithm and outcome scoring functions.

Multimedia Appendix 3 Characteristics of community-dwelling older participants.

Multimedia Appendix 4 Instances of personalized meal recommendations.

## Abbreviations

CDGI-E: China Elderly Dietary Guidelines Index
DDS: dietary diversity score
FoodKG: food knowledge graph
FRAIL: short 5-question assessment of fatigue, resistance, aerobic capacity, illnesses, and loss of weight
IoT: Internet of Things
MCC: multiple chronic condition

## Data Availability

The data sets used and analyzed during this study are not publicly available for data privacy concerns but may be available from the corresponding author on reasonable request.
