# Supplementary material for: Developing a Personalized Meal Recommendation System for Chinese Older Adults: Observational Cohort Study
Source: JMIR Form Res. 2024 May 30;8:e52170. doi: 10.2196/52170 (PMC11176883; doi:10.2196/52170)
Supplement: Multimedia Appendix 1 [file formative_v8i1e52170_app1.pdf]

Appendix 1: FoodKG data preparation

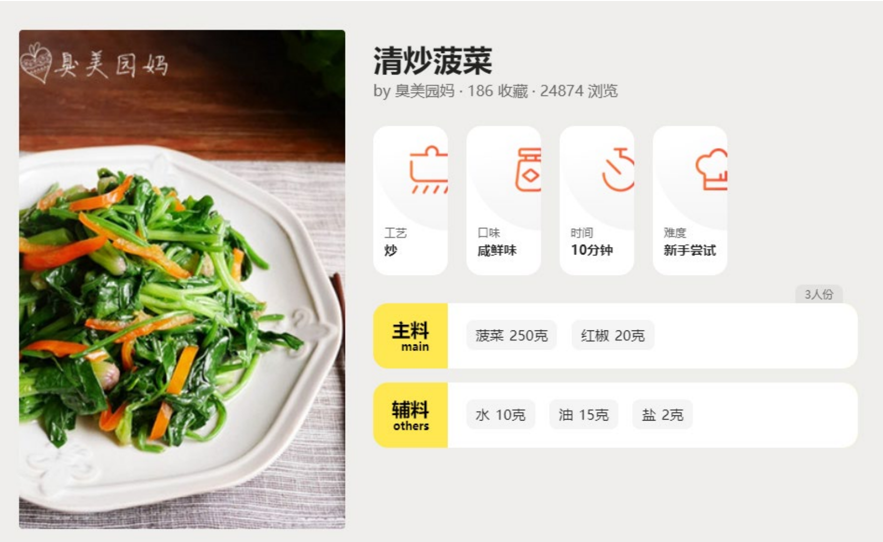

Website	Source	Name	Cooking method	Flavor	Time	Difficulty	Main ingredient	Seasoning
https://m.meishij.net/	Mei Shi Jie	Sautéed Spinach	Frying	Salty and fresh	10 minutes	Entry-level	Spinach: 200g<br>Red pepper: 20g	Water: 10g<br>Oil: 15g<br>Salt: 2g

Figure 1. Web page food data transition instance

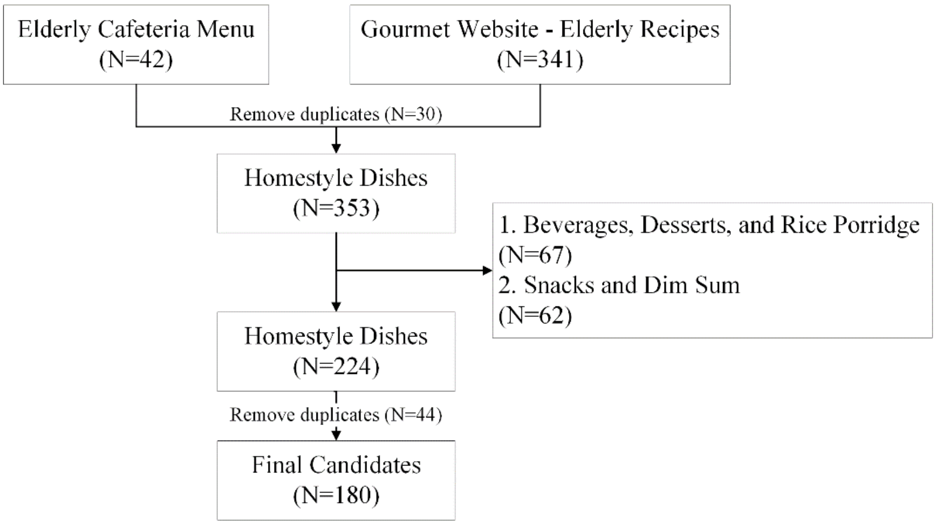

Figure 2. Dish finalizing flowchart

Table 1. Standards for dish composition

Category	Description
Vegetables	150g
Soup[a]	180g

Rice	150g
Fried rice	150g rice, 100g vegetables
Noodles with side dishes[b]	200g noodles, 50g side dishes
Porridge	300g
Sodium percentage	0.9±0.1(%)

[a]Soup ingredients calculated at 60g raw materials.

[b]Side dish ingredients to porridge ratio 1:5

Table 2. Representative cases for relation data

Relationship	Triple	Case Sentence from Corpus	Source of Corpus
Avoid	(Chronic Atrophic Gastritis, Avoid, Pickled Vegetables)	"Do not consume pickled and non-fresh foods"	Pocket Book of Geriatrics, Dietary Guidelines for the Elderly in China [1,2]
Avoid	(Hypertension/Diabetes/ Dyslipidemia/Stroke, Avoid, Fried)	"Elderly people with slower metabolic functions should pay attention to a light diet, avoiding high salt and fried foods"	Dietary Guidelines for the Elderly in China, Chinese Guideline on Healthy Lifestyle to Prevent Cardiometabolic Diseases[1,3]
Avoid	(Gout, Avoid, Pork Liver)	"Avoid high-purine animal products (animal offal, shellfish, concentrated meat soups and gravies, etc.)"	Guideline for primary care of gout and hyperuricemia (2019) [4]
Limit	(Gout, Limit, Purine)	"Daily dietary purine intake should be controlled under 200mg"	Guideline for primary care of gout and hyperuricemia (2019) [4]
Limit	(Diabetes, Limit, Eggs)	"No more than 4 eggs per week"	Dietary Guidelines for Type 2 Diabetic Patients in China [5]
Limit	(Diabetes, Limit, Cholesterol)	"Cholesterol intake should not exceed 300mg/d"	Dietary Guidelines for Type 2 Diabetic Patients in China [5]
Suitable	(Cancer, Suitable, Protein)	"Protein intake should exceed 1g/(kg·d)"	CSCO Nutrition in Cancer Patients [6]
Limit	(Stroke, Limit, Salt)	"Salt intake should not exceed 5g per day"	Dietary Guide for Stroke Patients [7]

Suitable	(Stroke, Suitable, Vegetables)	"Daily vegetable intake should be over 500 g"

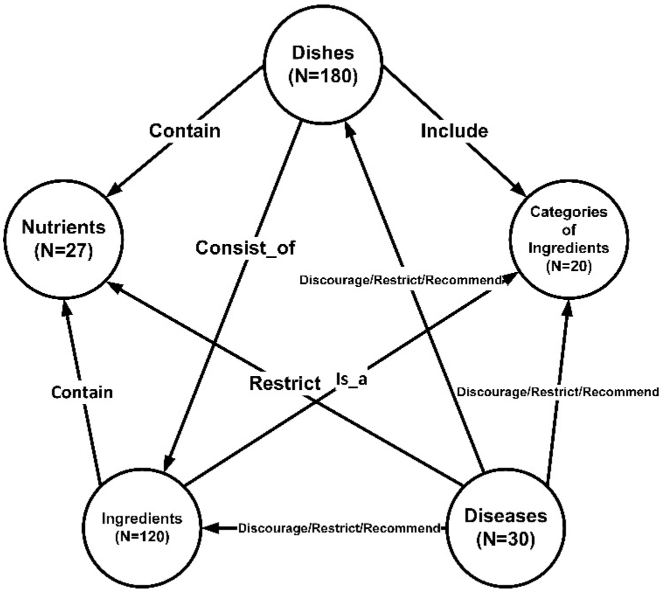

Figure 3. Food-KG schema

A 'dish' refers to any edible cuisine composed of various 'ingredients', which include raw materials and seasonings, thus establishing a 'consist_of' relation between 'dishes' and 'ingredients'. These 'ingredients' are grouped into different 'ingredient categories' in accordance with dietary guidelines, and relevant 'nutrients' are identified from food composition tables. 'Diseases' are incorporated as the final crucial concept.

Table 3. Definitions and examples of entities in Food-KG

Concept	Definition	Examples
Dish	A ready-to-eat prepared food item	Braised Pork with Bean Curd Sheets, Fish-flavored Shredded Pork, Seaweed Tofu Soup
Ingredients	All main ingredients, supplementary materials, and seasonings that compose a dish	Pork (rib meat), Bean Curd Sheets, Salt
Types of Ingredients	Categories to which ingredients belong	Grains and Cereals, Meat and Meat Products, Vegetables

Nutrients	Substances in food ingredients or dishes that can be absorbed and utilized by the human body to support life activities, including water, calories, macronutrients, major nutrients, trace nutrients, vitamins, etc.	Fats, Proteins, Vitamin C
Diseases	Common diseases in the elderly	Diabetes, Hypertension

Table 4. Attributes of entities in Food-KG

Entity Concept	Attribute Name	Attribute Value	Example
Dish	id_recipe	Recipe ID	Braised Pork@id_recipe:2
Dish	name_recipe	Recipe Name	Braised Pork@name_recipe: Braised Pork
Dish	flavor_recipe	Flavor of Recipe	Braised Pork@flavor_recipe: Soy Sauce Flavor
Dish	cook_recipe	Cooking Method of Recipe	Stir-fried Lettuce@cook_recipe: Stir-fry
Dish	cat_recipe	Category of Recipe	Braised Pork Ribs@cat_recipe: Pure Meat Dish
Ingredient	id_food	Ingredient ID	Chicken Leg@id_food:57
Ingredient	name_food	Ingredient Name	Chicken Leg@name_food: Chicken Leg
Ingredient Type	id_cat	Category ID	Meat and Meat Products@id_cat:3
Ingredient Type	name_cat	Category Name	Vegetable Class@name_cat: Vegetable Class
Nutrient	id_nutri	Nutrient ID	Protein@id_nutri:2
Nutrient	name_nutri	Nutrient Name	Protein@name_nutri: Protein
Disease	id_disease	Disease ID	Hypertension@id_disease:1
Disease	name_disease	Disease Name	Hypertension@name_nutri: Hypertension

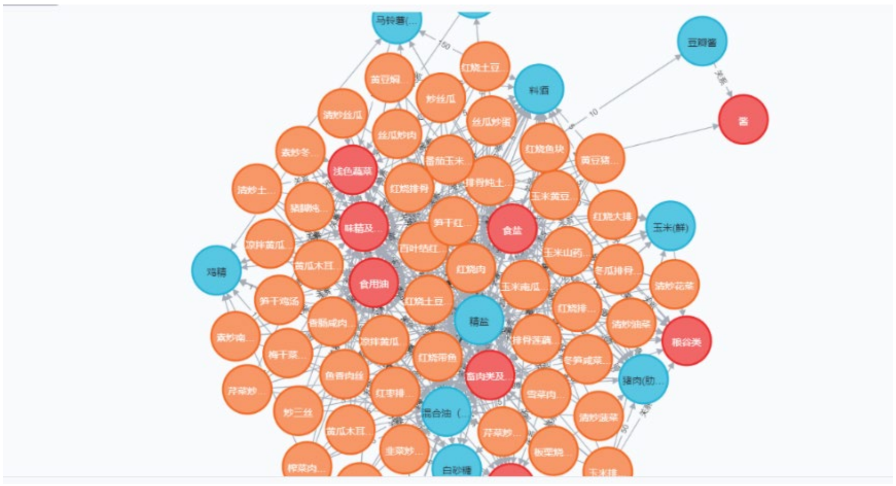

Figure 4. Food-KG screenshot

The entities of dishes (orange), ingredients (blue), and food category (red) act as nodes. An edge connects a pair of nodes and captures the interrelations, and the labels of edge further capture the meaning of the relations.

Table 5. Entities contained in elderly-targeted food knowledge graph

**Entity type**	**Count**
Dish	180
Ingredient	112
Ingredient category	20
Nutrient	27
Disease	30
Total	369

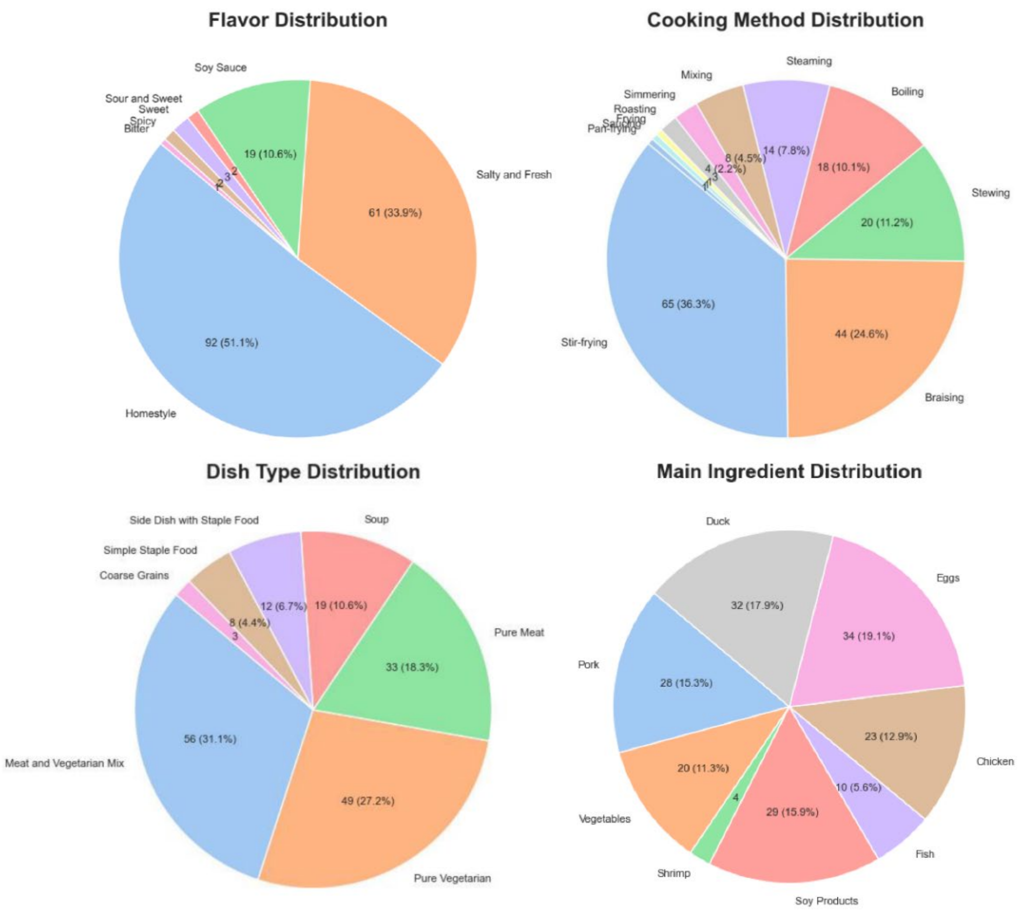

Figure 5. Distribution of included dishes' attributes (N=180)

Reference

[1] Chinese Nutrition Society. Dietary Guidelines for the Elderly in China (2016) [M]. Beijing: People's Medical Publishing House, 2018. ISBN/ISSN: 978-7-117-26998-8

[2] Liu Xiaohong, Zhu Minglei. Pocket Book of Geriatrics [M]. Beijing: People's Medical Publishing House, 2014. ISBN/ISSN: 978-7-117-18830-2/R · 18831

[3] Gu Dongfeng, Weng Jianping, and Lu Xiangfeng. Chinese Guideline on Healthy Lifestyle to Prevent Cardiometabolic Diseases [J]. Chinese Circulation Journal. 2020, 35(03): 209-230. doi:CNKI:SUN:ZGXH.0.2020-03-001.

[4] Chinese Medical Association, Chinese Medical Journals Publishing House, Chinese Society of General Practice, et al. Guideline for primary care of gout and hyperuricemia(2019) [J]. Chinese General Practice, 2020, 19(04)： 293-303. DOI:10.3760/cma.j.cn114798-20200328-00382

[5] Ge Sheng, Zhang Pianhong, Ma Aiqin, et al. "Dietary Guidelines for Type 2 Diabetic Patients in China" and Interpretation [J]. Acta Nutrimenta Sinica, 2017, 39(06): 521-529.DOI: 10.13325/j.cnki.acta .nutr.sin.2017.06.004

[6] Chinese Society of Clinical Oncology. Guidelines of Chinese Society of Clinical Ooncology (CSCO) Nutrition in Cancer Patients (2021 Edition) [M]. Beijing: People's Medical Publishing House, 2021. ISBN/ISSN: 978-7-117-28362-5

[7] WS/T 558—2017. Dietary Guide for Stroke Patients [S]. Beijing: National Health Commission of the People's Republic of China, 2017.
