# Supplementary material for: Developing a Personalized Meal Recommendation System for Chinese Older Adults: Observational Cohort Study
Source: JMIR Form Res. 2024 May 30;8:e52170. doi: 10.2196/52170 (PMC11176883; doi:10.2196/52170)
Supplement: Multimedia Appendix 2 [file formative_v8i1e52170_app2.pdf]

Multimedia Appendix 2

The Personalized Meal Recommendation Algorithm

Candidate dish generation

Candidate dishes generation starts with dish-related restraints stored in food KG and user profile information as input, and a rule-based reasoning is used to identify and exclude dishes containing inappropriate ingredients for elderly individuals. The first step ensures the recommendations meet elderly individuals' disease-related nutritional requirements. The second step is designed to meet personal dietary preferences. The targeted community-dwelling elders are divided into two groups depending on their available eating history data. Respective solutions targeting cold boot or hot boot are given as follows.

For those community-dwelling elders with eating history data, the Cosine similarities is used to ranking dish candidates among the most frequently selected dishes by contained ingredients.

Given the ingredients set $U = \{u_1, u_2, \dots, u_n, \dots, u_N\}$ and dish set $D = \{d_1, d_2, \dots, d_m, \dots, d_M\}$, we have the ingredient-dish matrix $R \in \mathbb{R}^{N \times M}$ as:

$$R = \begin{bmatrix} r_{1-1} & \cdots & r_{1-n} \\ \vdots & \ddots & \vdots \\ r_{M-1} & \cdots & r_{M-N} \end{bmatrix} \quad (1)$$

where r_{nm} denotes the quantity of ingredient *n* contained in a standard unit of dish *m*. All ingredients composing the dish *m* can be also represented as following vector:

$$I_m = (S_{1m}, S_{2m}, \dots S_{nm}, \dots, S_{Nm})^T \quad (2)$$

where *N* denotes the number of ingredients composing the dish *m*; S_{nm} denotes the quantity of ingredient *n* contained in a standard unit of dish *m*. Then, the Cosine similarity to measure the similarity between two dishes i and j in terms of the ingredients they are composed of are calculated by:

$$Similarity(i,j) = \frac{s_i \cdot s_j}{||s_i|| \cdot ||s_j||} = \frac{\sum_{k=1}^{K} s_{ki} \cdot s_{kj}}{\sqrt{\sum_{k=1}^{K} s_{ki}^2} \cdot \sqrt{\sum_{k=1}^{K} s_{kj}^2}} \quad (3)$$

where S_{ki} and S_{kj} denotes the quantity of ingredient *k* contained in dish *i* and dish *j*, *K* denotes the number of ingredients composing a dish. The more similar two dishes are, the larger $Similarity(i,j)$ will be.

To address the cold boot for a targeting elder individual who had no eating history, we identify a reference elder individual who shared the most similar user profile information. Since the features in user profile items are designed to be categorical, a Jaccard similarity is used to calculate the user profile similarities as follows:

$$Similarity(a_i, b_j) = J(A,B) = \frac{|A \cap B|}{|A \cup B|} = \frac{|A \cap B|}{|A|+|B|-|A \cap B|} \in [0,1] \quad (4)$$

where a_i,b_j denote two community-dwelling elder individuals i, and j, and A,B denote their respective three-dimension characteristics represented in user profile.

Assuming that the most similar pairs of elderly individuals shared the same favorite dish (most highly frequently selected), the previous candidate dish filtering loop is subsequently executed. **Algorithm 1** shows the pseudocode of candidate dish filtering.

Algorithm 1 Candidate dish generation algorithm

Input: Dish-related data $D = \{D_{ingredient}, D_{foodgroup}, D_{attribute}\}$; User profile data $U = \{U_{health}, U_{surveyPref}, U_{eatHistory}\}$
Output: Recommended dish set R
Extract disease-related restrictions $R_{disease}$ from U_{health}.
Filter dishes violating $R_{disease}$ using D, generating candidate dishes C.
if $U_{eatHistory}$ is available **then**
    Identify TopN dishes P from $U_{eatHistory}$.
else
    Calculate Jaccard similarity J based on $U_{surveyPref}$ by equation (1).
    Select most similar individual I with $U_{eatHistory}$ as reference.
    Identify TopN dishes P from $I_{eatHistoryy}$.
end if
for each dish d_a in P **do**
    Extract ingredient amounts $I_a = \{S_{a1}, S_{a2}, ..., S_{aN}\}$ for d_a (See equation (2)).
    for each dish d_b in C **do**
        Extract ingredient amounts $I_b = \{S_{b1}, S_{b2}, ..., S_{bN}\}$ for d_b.
        Calculate Cosine similarity S between I_a and I_b by equation (3).
    end for
    Select TopK dishes K in C based on S.
end for
Finalize R by adding K and removing P.

Algorithm 1. Candidate dish generation algorithm

Combo meals recommendation

The combo meal generation procedure is designed as a three-step procedure. Firstly, assuming that the recommendation is only for lunch meals and considering a daily energy intake allocation of 3:4:3 for three meals, several dishes of different types are combined to create the initial set of combo meal candidates. Secondly, any combo meals that did not align with the user's nutritional constraints are removed, considering information on nutrients, food categories, and ingredients contained in the combos. Thirdly, the top N combo meals are generated as final recommendation outputs by ranking their nutrient adequacy based on the number of nutrients that meet the recommendations outlined in the Chinese elderly nutrition guidelines. The overall recommendation process can be formulated as:

$$A_i = \sum_{m=1}^{M} a_{im} \tag{5}$$

$$g_M(i) = \begin{cases} 1, & A_i \in [A_{lower}, A_{upper}] \\ 0, & otherwise \end{cases} \tag{6}$$

$$f(M) = \sum_{i=1}^{I} g_M(i) \tag{7}$$

$$R\% = 100\% \cdot \frac{f(M)}{I} \tag{8}$$

Specially, given I nutrients, a_{im} is the content of the ith nutrient in the jth dish, and A_i indicates the total content of the ith nutrient in the recommended package (**Eqn. 5**); S_i is the standard intake of the nutrient, and the recommended intake of each nutrient can be an interval, and according to whether or not the actual content of nutrients is in the recommended interval, we can get the segment function (**Eqn. 6**); the objective function of all Nutrient objective function for all nutrients can be summed up to get the objective function of the whole package, that is, the number of nutrients that meet the standard (**Eqn.** 7); the ratio of the amount of nutrients that meet the standard to the number of total nutrients can be expressed as the rate of nutrient compliance of the recommended package (**Eqn. 8**). **Algorithm 2** provided the pseudocode of personalized lunch combo generation.

Algorithm 2 Combo meals recommendation algorithm

Input: Recommended dish set R; User profile data $U = \{U_{health}\}$
Output: Recommended meal set M
Divide R into 5 categories: animal-derived dishes AD, vegan dishes VD, omnivorous dishes OD, staples ST, and soups SP.
Pair dishes in AD and VD to generate set $P1$.
Pair dishes in OD to generate set $P2$.
Pair soups with dish combinations in sets $P1$ and $P2$, generating sets $P3$ and $P4$.
Pair staples with dish combinations in sets $P1$, $P2$, $P3$, and $P4$, generating sets $P5$, $P6$, $P7$, and $P8$.
Pair animal-derived dishes with complete dishes to generate set $P9$.
Pair vegan dishes with complete dishes to generate set $P10$.
Pair omnivorous dishes with complete dishes to generate set $P11$.
Combine sets $P5$, $P6$, $P7$, $P8$, $P9$, $P10$, $P11$ into a set of recommended meal candidates MC.
Extract disease-related nutrient, ingredient, and ingredient type intake requirements from U_{health}.
for each meal m in MC **do**
    Calculate nutrient adequacy by equations (4) to (7).
end for
Filter out meal candidates in MC that violate the disease-related restrictions.
Extract the TopK meal candidates to finalize the recommended meal set M.

Algorithm 2. Combo meals recommendation algorithm

China Elderly Dietary Guidelines Index (CDGI) Scoring Function

CDGI is calculated by accumulating weighed points for ingredients at different recommendation levels in relevant to elderly dietary guideline.

$$Score_{CDGI} = \sum_{i=1}^{N} f(n_i) \tag{9}$$

$$f(n)_{substantial} = \alpha \cdot \begin{cases} \frac{2n}{c_{max}+c_{min}}, & n < \frac{c_{max}+c_{min}}{2} \\ 1, & n \geq \frac{c_{max}+c_{min}}{2} \end{cases} \tag{10}$$

$$f(n)_{sufficient} = \alpha \cdot \begin{cases} \frac{n}{c_{min}}, & n < c_{min} \\ 1, & c_{min} \leq n \leq c_{max} \\ 1 - \frac{n-c_{max}}{c_{max}}, & n > c_{max} \end{cases} \tag{11}$$

$$f(n)_{restricted} = \alpha \cdot \begin{cases} 1, & n < c_{max} \\ 1 - \frac{n-c_{max}}{c_{max}}, & n \geq c_{max} \end{cases} \tag{12}$$

Diet diversity score (DDS) Scoring Function

DDS is measured by total counts out of 9 ingredient categories.

$$Score_{DDS} = \sum_{i=1}^{F} \mathbf{1}_{f_i > 0} \tag{13}$$
