# Supplementary material for: Developing a Personalized Meal Recommendation System for Chinese Older Adults: Observational Cohort Study
Source: JMIR Form Res. 2024 May 30;8:e52170. doi: 10.2196/52170 (PMC11176883; doi:10.2196/52170)
Supplement: Multimedia Appendix 3 [file formative_v8i1e52170_app3.pdf]

Multimedia Appendix 3

Table 1. Characteristics of the community-dwelling older participant (N=96)

Item	Feature	Number	Percentage (%)
Sex	Male	51	53.12
	Female	45	46.88
BMI	≤19.9	2	2.08
	20.0-26.9	66	68.75
	≥27.0	27	28.12
Age	60-64	14	14.58
	65-79	66	68.75
	≥80	16	16.67
Monthly Household Income Per Capita (CNY)	≤2000	7	7.29
	2001-4000	71	73.96
	4001-6000	17	17.71
	≥6001	1	1.04
Living Situation	Alone	15	15.62
	With spouse	36	37.5
	With children, relatives, or friends	45	46.88
Physical Activity Level	Low	69	71.87
	Moderate	27	28.13
Risk of Malnutrition	Normal	87	90.63
	At risk	9	9.37
Risk of Frailty	No risk	76	79.17
	Pre-frailty	20	20.83
Diagnosed Diseases Classification	Cardiovascular diseases	83	86.46
	Musculoskeletal diseases	41	42.71
	Endocrine and metabolic diseases	26	86.46
	Digestive system diseases	18	18.75
	Eye diseases	18	86.46
	Hearing disorders	7	7.29
	Cancer	4	86.46
	Neurological disorders	3	3.13

	Urinary system diseases	2	86.46
	Respiratory system diseases	2	2.08
	Prostate diseases	1	86.46
	Mental and psychological disorders	1	1.04
Cooking Method Preference	Stir-frying	53	55.21
	Steaming	34	35.42
	Boiling	12	12.5
	Frying	5	5.21
	Stewing	2	2.08
Preferred Cooking Oil	Other vegetable oils	94	97.92
	Sesame oil	1	1.04
	Other animal fats	1	1.04
	Lard	1	1.04
Flavor Preference	Light	59	61.46
	Salty	33	34.38
	Sweet	4	4.17
	Spicy	3	4.17
	Raw and cold	1	1.04
	Unknown	2	2.08
Staple Food Preference	Rice	80	83.33
	Half rice, half flour	12	12.5
	Flour	3	3.13
	Mixed grains	1	1.04
