# Supplementary material for: Developing a Personalized Meal Recommendation System for Chinese Older Adults: Observational Cohort Study
Source: JMIR Form Res. 2024 May 30;8:e52170. doi: 10.2196/52170 (PMC11176883; doi:10.2196/52170)
Supplement: Multimedia Appendix 4 [file formative_v8i1e52170_app4.pdf]

Multimedia Appendix 4. Instances of personalized meal recommendations

Table 1. Recommended and Automously planned meals for community-dwelling older adults with tracked eating history

Date	Monday	Tuesday	Wednesday	Thursday	Friday
Autonomously selected Dishes in Combo Meal	Rice	Rice	Rice	Rice	Rice
	Steamed Pork Patty with Mushrooms	Sliced Cold Chicken	Pigs' Trotters Stewed with Soybeans	Steamed Pork Patty with Mushrooms	Bean Curd Roll with Meat
	Potherb Mustard with Tofu	Stir-fried Greens	Stir-fried Cabbage, Tofu, and Pork	Braised Vegetarian Chicken	Stir-fried Cabbage, Tofu, and Pork
CDGI-E	22.36	29.85	23.96	23.7	23.12
DDS	3	3	4	4	4
Recommended Dishes in Combo Meal	Stir-fried Carrot with Beef	Roasted Purple Sweet Potato	Rice	Braised Pork with Chestnuts	Roasted Pumpkin
	Braised Potato with Chicken	Steamed Meat Patty	Stir-fried Leek with Eggs	Stir-fried Potato with Meat	Steamed Egg Custard
	Seaweed Tofu Soup	Stir-fried Rape with Tofu	Stuffed Tofu with Meat	Roasted Purple Sweet Potato	Stir-fried Cabbage with Tofu
	Rice	Seaweed Tofu Soup	Corn and Soybean Rib Soup	Steamed Bun with Mushroom and Meat	Tomato and Corn Rib Soup
CDGI-E	32.35	30.64	31.61	33.08	29.73
DDS	6	4	4	6	4

Table 2. Recommended meals for community-dwelling older adults without tracked eating history

Date	Monday	Tuesday	Wednesday	Thursday	Friday
Recommended Dishes in Combo Meal	Stir-fried Potato with Meat	Roasted Sweet Potato	Steamed Meat Patty	Rice	Steamed Egg Custard
	Braised Pork with Carrot	Sliced Pork with Garlic Sauce	Stir-fried Spinach	Steamed Chicken with Chili Sauce	Stir-fried Shredded Potato
	Seaweed Tofu Soup	Stir-fried Leek with Dried Tofu	Mushroom Chicken Soup	Stir-fried Cabbage with Tofu	Tomato Corn Rib Soup
	Rice	Seaweed Tofu Soup	Rice	Tomato Egg Soup	Steamed Bun with Stuffed Carrot and Meat
CDGI-E	32.49	30.25	28.65	29.81	30.3
DDS	6	5	4	6	4
